# Down-regulation of DNMT3b in PC3 cells effects locus-specific DNA methylation, and represses cellular growth and migration

**DOI:** 10.1186/1475-2867-8-13

**Published:** 2008-09-17

**Authors:** Ahmed Yaqinuddin, Sohail A Qureshi, Romena Qazi, Farhat Abbas

**Affiliations:** 1 Department of Biological & Biomedical Sciences, The Aga Khan University, Stadium Road, Karachi 74800, Pakistan; 2 Department of Pathology & Microbiology, The Aga Khan University, Stadium Road, Karachi 74800, Pakistan; 3 Department of Surgery, The Aga Khan University, Stadium Road, Karachi 74800, Pakistan

## Abstract

**Background:**

Aberrations in DNA methylation patterns promote changes in gene expression patterns and are invariably associated with neoplasia. DNA methylation is carried out and maintained by several DNA methyltransferases (DNMTs) among which DNMT1 functions as a maintenance methylase while DNMT3a and 3b serve as de novo enzymes. Although DNMT3b has been shown to preferentially target the methylation of DNA sequences residing in pericentric heterochromatin whether it is involved in gene specific methylation remains an open question. To address this issue, we have silenced the expression of DNMT3b in the prostate-derived PC3 cells through RNA interference and subsequently studied the accompanied cellular changes as well as the expression profiles of selected genes.

**Results:**

Our results demonstrate that DNMT3b depletion results in increased apoptosis and reduced migration of PC3 cells compared to the untransfected control cells. Reduced DNMT3b expression resulted in hypomethylation of retinoblastoma (Rb), retinoic-acid receptor β (RAR-β), and adenomatous polyposis coli (APC) gene promoters, and also culminated in increased expression of *CDKN3 *and *cytochrome b5*. Although DNMT3b silenced cells were found to have reduced growth and migratory potential, there was no apparent changes in their invasive ability compared to the parental PC3 cell line.

**Conclusion:**

Our findings reveal that DNMT3b preferentially targets certain gene promoters in PC3 cells and that its depletion significantly reduces growth and migration of PC3 cells.

## Background

Mutations, chromosomal translocations and dysfunctional epigenetic mechanisms alter gene expression patterns which invariably culminate in neoplasia [1]. DNA methylation represents one type of epigenetic modification which influences gene expression. Given that hypomethylation upregulates gene expression whereas hypermethylation has the opposite effect, unprogrammed DNA modifications that either repress tumor suppressor gene expression or activate oncogene expression are likely to promote uncontrolled cellular proliferation [2].

DNMT1, DNMT3a and DNMT3b are the three main DNA methyltransferases responsible for establishing DNA methylation patterns during development as well as maintaining them in differentiated cells [3]; DNMT2 represents a fourth member of the family, but its involvement in promoting DNA methylation remains unclear [4]. DNMT1 fully methylates hemimethylated CpG residues in DNA. As a maintenance methylase DNMT1 associates with proliferating cell nuclear antigen (PCNA) and is found at the DNA replication forks during S-phase. Recently, a novel protein UHRF1 was identified which binds to hemimethylated DNA and recruits DNMT1; this protein co-localizes with DNMT1 at replication foci during the S-phase of cell cycle [5].

DNMT3a and DNMT3b consist of 908 and 859 amino acids, respectively, and function as *de novo *DNA methylases [6]. Both enzymes are abundant in embryos, but their expression in differentiated somatic cells which do not require new methylation is very low [7-10]. Accumulation of 5-methylcytosine epimutations by aberrant DNMT1, DNMT3a and 3b expression has been found to silence tumor suppressor genes including APAF, p16 and RASSF1A [11-13]. Many primary tumors have been found to contain high levels of these enzymes [14,15].

The essential role of DNMT1, DNMT3a and DNMT3b on embryonic development is well known but how dependent these proteins are on each other for function in different types of normal as well as cancerous cells remains elusive. Cell-based, biochemical genetic studies have revealed that a complex interplay exists between these enzymes. The picture that has emerged for DNMT3b in particular is especially conflicting because its depletion from different human cells has been found to produce extremely variable effects [16-18]. In this study we have silenced the expression of DNMT3b in prostate cancer derived PC3 cells, which have not been studied previously, and determined its consequences on gene specific methylation and expression as well as on cellular processes such as apoptosis and cell migration.

## Methods

### Cell Culture

PC3 cell line was obtained from American Type Culture Collection (ATCC, Manassas, VA) and maintained in RPMI with 10% fetal bovine serum, 2 mM L-glutamine, and 100 U/ml penicillin + streptomycin sulfate (Invitrogen).

### Transfections

Plasmids psiRNA™ is a family of expression vectors designed to generate shRNAs from RNA III promoter (Invivogen Inc.). PC3 cells were transfected with a mock plasmid as well as a validated plasmid expressing shRNA that specifically targets DNMT3b mRNAs to produce control and psiDNMT3b cell lines, respectively. psiDNMT3b harbors a GFP:zeocin fusion cassette for selection and assessing transfection efficiency. Transfections were carried out using 3 μl of Fugene-6 (Roche Inc) and 1 μg of plasmid DNA. After 48 hours of transfection, cells were trypsinized, diluted 1:15 and placed in a 50 μg/ml zeocin containing medium, which was replaced with fresh medium every 3 days. After 3 weeks, resistant colonies (~200–300 clones) were trypsinized, combined in pools, cultured in selection medium, and expanded into cell lines.

### PCR

RT-PCR was performed to validate silencing of the target gene in PC3 cells. Total RNA was extracted from control as well as silenced PC3 cell line using Trizol (Invitogen). Reverse transcription was carried out using First-Strand Reverse Transcription kit (Invitogen) and 1 μg of total RNA. Subsequently, 2 μl of RT reaction was used in PCR. Primer sets corresponding to DNMT3b gene were used with sequences as previously described [19]. Semi-quantitative RT-PCR for DNMT3b was carried out using the following thermocycling conditions: 94°C for 2 minutes (1 cycle); 94°C for 30 seconds, 60°C for 1 minutes, 72°C for 1 minute (25 cycles); and 72°C for 7 minutes. Primer sets for CDKN3 and cytochrome b5 were purchased from Superarray Biosciences. Thermocycling conditions for amplification of CDKN3 and cytochrome b5 were: 94°C for 2 minutes (1 cycle); 94°C for 30 seconds, 55°C for 40 seconds, 72°C for 1 minute (25 cycles); and 72°C for 7 minutes.

### Western Blotting

Extracts from control and DNMT3b silenced cell lines were prepared by resuspending cell pellets (~2 × 10^5 ^cells) in sample loading buffer [0.125 M Tris-HCl (pH 6.8), 4% SDS, 20% glycerol and 10% 2-mercaptoethanol]. Approximately 50 μg of the protein was separated by electrophoresis on a 10% SDS-PAGE and the gel contents transferred to nitrocellulose membrane (Amersham). After blocking overnight at 4°C in 5% nonfat dry milk prepared in TBST, the membrane was probed with a 1:1000 dilution of DNMT3b, caspase 3 and MCM2 antibodies (Santa Cruz Biotech) for 1–2 hours at room temperature. Membranes were washed with TBST, incubated with the appropriate HRP-conjugated secondary antibody and subsequently developed with the enhanced chemiluminescence (ECL) western blotting system (Amersham). β-actin was included as the internal loading control and was detected using a specific antibody (Santa Cruz Biotechnology).

### MTT Assay

Cellular proliferation was measured by the 3-(4,5-dimethyl thiazol-2-yl)-2,5-diphenyl tetrazolium bromide (MTT) proliferation assay kit (ATCC) according to the manufacturer's instructions. Briefly, 10^4 ^cells were seeded in 96-well plates and cultured in 5% FCS for 24 hours. Before testing, 10 μl of MTT labeling reagent (5 mg/mL MTT) was added to cells and the mixture incubated for a further 4 hours at 37°C. 100 μl of solubilizing reagent was then added and the plate incubated overnight at 37°C to dissolve formazan crystals. Absorbance was measured at OD_595 _in a Chameleon multilabel detection platform (Hidex Inc.). Each assay was carried out in triplicate and each experiment was repeated at least twice.

### Acridine Orange and Ethidium Bromide (AO/EB) Staining

Apoptosis was measured by AO/EB staining as described previously with some modifications [20]. Briefly 25 μl of cell suspension (1–2 × 10^5 ^cells) was added to 25 μl of 1:1 AO/EB solution (both AO and EB prepared at 100 μg/ml in PBS) and loaded on a hemocytometer under a cover slip. Cells were counted on a grid using visible light and then dead cells (stained orange with ethidium bromide) were counted under fluorescence. Student T-test was performed to assess the statistical significance of obtained data.

### Boyden Chamber Invasion Assay

Boyden chamber invasion assay was performed as described [21] with some modifications. A fixed number of cells (5 × 10^4 ^cells/ml) in serum-free medium were placed in the upper chamber of the Boyden chamber pre-coated with Matrigel (polyvinyl pyrrolidone-free polycarbonate filter with 8-μm pore size) inserts (BD Pharmingen, San Diego, CA). The lower chamber contained serum-free media with epidermal growth factor (50 ng/ml). Cells were incubated for 24 hours, after which medium was removed and the upper surface of the invasion chamber insert was scrubbed with a cotton swab thrice in each direction. The membrane was removed from the invasion chamber insert with a scalpel, was fixed with 100% methanol and stained with Giemsa Wright stain. Cells were counted at 200× magnification in five different random fields by two independent observers. Student's T-test was performed to assess statistical significance of obtained invasion data.

### Cell Migration Assay

Stably transfected cells were sub-cultured in 6-well plates and incubated in RPMI with 10% FBS. Confluent monolayer cells were starved for serum and growth factors in RPMI for 24 h and beeline or river (1 mm thick) was created using a 10 μl micropipette tip.

Cells were washed and incubated in serum free medium. Cell migration was monitored at 12-hr interval and photographed.

### Methylation-Specific PCR (MSP)

Genomic DNA was extracted from control and psiDNMT3B transfected cells by using DNA extraction kit (Qiagen, Hilden). 2 μg of DNA was subjected to bisulphite modification using Methyl-Easy bisulphite modification kit (Human Genetic Signatures, Sydney, Australia). Bisulphite converted DNA was then amplified with two sets of primers (methylated and unmethylated) for the following genes RASSF1a, APC, RAR-beta, RB1, hMLH1, Survivin, DAPK, BRCA, TIMP3, VHL, p16, PTEN, CDH1, CASP8, hTERTc, and RASSF1c; DNA sequences of primers employed for PCR are listed in Table-1. PCRs were carried out on Master Cycler (Eppendorf) using the following conditions: 95°C for 10 minutes, followed by 40 cycles of 95°C for 30 seconds, 55°C for 30 seconds, and 72°C for 1 minute followed by a final extension at 72°C for 7 minutes. Each PCR was carried out in 25 μl reaction volume containing 1× AmpliTaq Gold PCR buffer II (Applied Biosystems), 1 unit AmpliTaq Gold polymerase (Applied biosystems), 1 μM forward primer, 1 μM reverse primer, 0.25 mM dNTPs each,1.5 mM MgCl_2 _and 2 μl of DNA. Bisulphite converted Sss I methylase-treated WBC DNA served as positive control.

**Table 1:** DNA sequences of all primers used in methylation sensitive PCR

**GENE**	**METHYLATED PRIMER SEQUENCE**	**UNMETHYLATED PRIMER SEQUENCE**
RASSF1A	F 5' GTGTTAACGCGTTGCGTATC 3'	F 5'TTTGGTTGGAGTGTGTTAATGTG 3'
	R 5' AACCCCGCGAACTAAAAACGA 3'	R CAAACCCCACAAACTAAAAACAA 3'
Survivin	F 5' GGCGGGAGGATTATAATTTTCG 3'	F 5' GGTGGGAGGATTATAATTTTTG 3'
	R 5' CCGCCACCTCTACCAACG 3'	R 5' CCACCACCACCACCTCTAC 3'
DAPK1	F 5'GGATAGTCGGATCGAGTTAACGTC 3'	F 5'GGATAGTTGGATTGAGTTAAYGTC 3'
	R 5' CCCTCCCAAACGCCGA 3'	R 5' CAAATCCCTCCCAAACACCAA 3'
APC	F 5' TATTGCGGAGTGCGGGTC 3'	F 5' GTGTTTTATTGTGGAGTGTGGGTT 3'
	R 5' TCGACGAACTCCCGACGA 3'	R 5' CCAATCAACAAACTCCCAACAA 3'
RAR-β	F 5' TCGAGAACGCGAGCGATTCG 3'	F 5' TTGAGAATGTGAGTGATTTGA 3'
	R 5' GACCAATCCAACCGAAACGA 3'	R 5' AACCAATCCAACAAAACAA 3'
hLMH1	F 5'ACGTAGACGTTTATTAGGGTCGC 3'	F 'TTTTGATGTAGATGTTTTATTAGGGTTGT 3'
	R 5'CCTCATCGTAACTACCGCG 3'	R 5'ACCACCTCATCATAACTACCCACA 3'
RB1	F 5'GGGAGTTTCGCGGACGTGAC 3'	F 5'GGGAGTTTTGTGGATGTGAT 3'
	R 5'ACGTCGAAACACGCCCCG 3'	R 5'ACATCAAAACAACCCCA 3'
BRCA	F 5'GGTTAATTTAGAGTTTCGAGAGACG 3'	F 5' GGTTAATTTAGAGTTTTGAGAGATG 3'
	R 5' TCAACGAACTCACGCCGCGCAATCG 3'	5' TCAACAAACTCACACCACACAATCA 3'
TIMP3	F 5' CGTTTCGTTATTTTTTGTTTTCGGTTTC 3'	F 5' TTTTGTTTTGTTATTTTTTGTTTTTGGTTTT 3'
	R 5' CCGAAAACCCCGCCTCG 3'	R 5' CCCCCAAAAACCCCACCTCA 3'
VHL	F 5' TGGAGGATTTTTTTGCGTACGC 3'	F 5' GTTGGAGGATTTTTTTGTGTATGT3'
	R 5' GAACCGAACGCCGCGAA 3'	R 5' CCCAAACCAAACACCACAAA 3'
p16	F 5' TTATTAGAGGGTGGGGCGGATCGC 3'	F 5' TTATTAGAGGGTGGGGTGGATTGT 3'
	R 5' ACCCCGAACCGCGACCGTAA 3'	F 5' CAACCCCAAACCACAACCATAA 3'
PTEN	F 5' GGTTTTTCGAGGCGTTCG 3'	F 5' TGGTTTTTTGAGGTGTTTG 3'
	R 5' CGCCTCACAACGACTCAACT 3'	R 5' TTCCATCATAACTACAACTTCCA 3'
CDH1	F 5' GTGGGCGGGTCGTTAGTTTC 3'	F 5' GGTGGGTGGGTTGTTAGTTTTGT 3'
	R 5' CTCACAAATACTTTACAATTCCGACG 3'	R 5' AACTCACAAATCTTTACAATTCCAAC 3'
CASP8	F 5' TAGGGGATTCGGACATTGCGA 3'	F 5' TAGGGGATTTGGAGATTGTGTA 3'
	R 5' CGTATATCTACATTCGAAACG 3'	R 5' CCATATATATCTACATTCAAAACAA 3'
hTERTc	F 5' GACGTAAAGTTTTTTTCGGACG 3'	F 5' GTAAAGATGTAAAGTTTTTTTTGGATG 3'
	R 5' ACCCGATACGCTACCGAACG 3'	R 5' CCACAACCCAATACACTACCA 3'
RASSF1c	F 5' AGTTTGGATTGTCGGTTTCG 3'	F 5' GGAGTTTGGATTGTTGGTTTTG 3'
	R 5' TCACAAACCCCACCTACCAC 3'	R 5' CACCCCCAAAAATAACCTCAT 3'

DNA sequences of methylated and unmethylated set of primers of 16 tumor suppressor genes studied by methylation-specific PCR.

### Genechip Expression Arrays

Prostate cancer specific oligo-GEarray (SuperArray Biosciences) was used for gene expression profiling. Briefly, total RNA was extracted from psiDNMT3b and mock transfected cells by Arraygrade™ Total RNA isolation kit (SuperArray Biosciences). Integrity of RNA was assessed by running 1 μg of RNA on denaturing agarose gel. Truelabeling AMP-2.0 kit (SuperArray Biosciences) was used for synthesizing biotin labeled cRNA. Labeled probe was hybridized to prostate cancer specific oligoGEarray. Arrays were developed using chemiluminescent detection kit and X-ray film was used to capture images. For analysis, the X-ray film pattern was scanned and loaded on the GEarray expression Analysis Suite – a web based software from Superarray. The relative expression of 288 genes was assessed using this software and the respective signals normalized against the spot intensities of β-actin, GAPDH and ribosomal proteins on the membrane.

## Results

**Figure 1:**
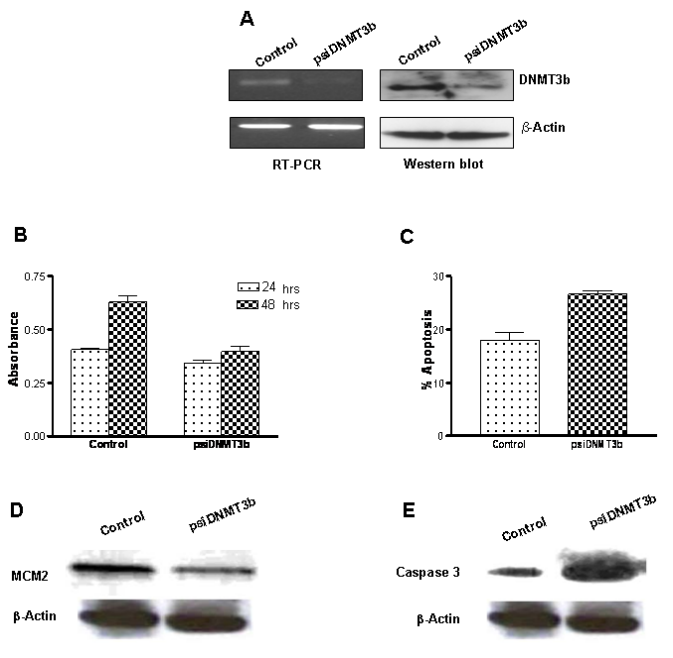
**Silencing of DNMT3b expression in PC3 cells and validation of cell lines.** A. Total RNA as well as protein was extracted from mock (control), and psiDNMT3b transfected PC3 cells and subjected to semi-quantitative RT-PCR analysis and Western blotting. β-actin was used as loading control. B. Affect of DNMT3b silencing on cell proliferation. Cells were grown and their respective rate of proliferation was measured by MTT assay after 24 and 48 hours. Absorbance was measured at 595 nm. C. Apoptosis was studied quantitatively by ethidium bromide and acridine orange staining of control and DNMT3b silenced cells. D. Expression analysis of MCM2. Total protein from mock (control), and psiDNMT3b transfected PC3 cells was subjected to Western blotting to determine expression of MCM2. β-actin was used as loading control. E. Expression analysis of caspase 3. Total protein from mock (control), and psiDNMT3b transfected PC3 cells was subjected to Western blotting to determine expression of caspase 3. β-actin was used as loading control.

DNMT3b deficiency in colon cancer cells results in a very nominal 3% reduction in DNA methylation levels and does not appear to alter the methylation status of any genes [17]. To determine whether depletion of DNMT3b in PC3 cells has the same effect, RNA interference was employed to reduce its expression. This was carried out by stably transfecting a plasmid from which a shRNA is synthesized, processed and supposedly incorporated into an RNA-induced silencing complex (RISC) that specifically targets DNMT3b mRNA; this plasmid also expresses GFP which permitted facile visual inspection of cells through epifluorescence microscopy. After maintaining transfected cells in zeocin-containing medium for two weeks, DNMT3b mRNA and protein levels were determined using RT-PCR and Western blotting. Such analysis revealed that even though 100% of the cells were GFP-positive, expression of DNMT3b was reduced by approximately 75% at the protein level relative to the mock-transfected parental cells (Figure-1A). Additional attempts at further reducing the DNMT3b expression did not prove successful. Importantly, DNMT3b down-regulation did not impact DNMT1 expression in the engineered PC3 cells as gauged by RT-PCR and Western blot analyses (data not shown).

Phenotypic changes in PC3 cells should not be observed if other DNMTs are able to compensate for DNMT3b loss. To assess whether any phenotypic changes were brought about by depletion of DNMT3b, we compared cellular processes such as proliferation, apoptosis, invasion and migration in the engineered cells with the control mock-transfected PC3 cells. Proliferation analyses, as carried out by the MTT assay, revealed that cells with reduced levels of DNMT3b grew significantly more poorly than the control cells after 24 and 48 hours (p-value, < 0.0002, and < 0.0001 respectively; Figure-1B). The expression of MCM2, a proliferation marker, was determined in both cell lines and showed more than 50% reduction in DNMT3b depleted cells as compared to control cells (Figure-1D). This growth defect was attributed to increased apoptosis which in DNMT3b deficient cells was found to be approximately 35% more than the control cells (p-value < 0.0011; Figure-1C). Interestingly, caspase-3 expression was found to be increased two-fold in DNMT3b silenced cells which exhibited enhanced apoptosis and poor growth (Figure-1E).

**Figure 2:**
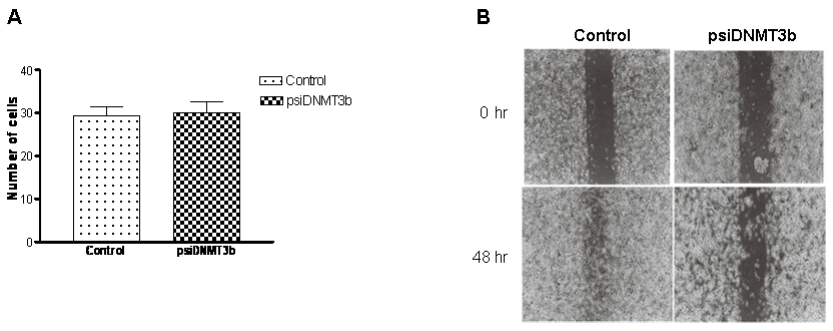
**Cellular invasion and migration assays.** A. Boyden chamber invasion assays were carried out on control and DNMT3b silenced cells and the number of cells invading matrigel was determined. B. Wound healing migration assays were carried out as described. Panels show wounds in control, and DNMT3b depleted cells at 0 and 48 hours.

Boyden-chamber assays revealed that the invasive potential of control and DNMT3b silenced PC3 cells was equivalent (Figure-2A). Invasion is a two-step process comprised of migration and membrane (matrigel in Boyden chamber assays) breakdown; to be invasive cells must be able to execute both steps competently. Given that DNMT3b deficient cells were no different from control cells in terms of their invasive potential we reasoned that they might be compromised with their ability to migrate. Towards that end, migratory potential of both cell lines was compared using the wound healing assay. This experiment demonstrated that down-regulation of DNMT3b in PC3 cells significantly reduced their ability to trespass through a trough even after 48 hours (Figure-2B).

**Figure 3:**
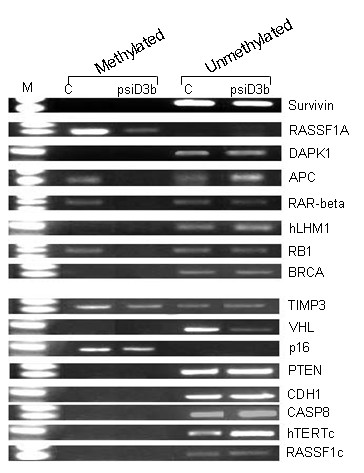
**Methylation specific PCR analyses of RASSF1a, APC, RAR-β, RB1, hMLH1, Survivin, DAPK, BRCA, TIMP3, VHL, p16, PTEN, CDH1, CASP8, hTERTc, and RASSF1c genes in mock and psiDNMT3b transfected cells.** Presence of PCR product indicates methylated (lane M) or unmethylated (lane U) alleles.

Previous reports have shown that silencing of DNMT3b does not appear to affect DNA methylation of any of the genes in cells derived from colon cancer. To determine whether the same affect of DNMT3b down-regulation is observed in prostate derived PC3 cells, we evaluated the methylation status of 16 tumor suppressor gene promoters using methylation-specific PCR (MSP). For this, genomic DNA from control as well as DNMT3b silenced cells was isolated, 5-methylcytosine residues chemically converted to uracil by bisulfite treatment, and subsequently employed this DNA as template in PCR reactions with methylation-senstitive as well as insensitive primer pairs. To ascertain that the employed primer sets can reliably distinguish methylated annealing sites from those which are unmethylated, genomic DNA was isolated from white-blood cells and one-half of it methylated to saturation with Sss1 methylase while the other half was used without any processing. Data from this experiment demonstrated clearly that in the diploid PC3 genome both promoters of the *RASSF1A *and *p16 *genes are hypermethylated and remain that way even after DNMT3b expression diminished (Figure-3). In contrast, both copies of genes encoding *survivin, DAPK1, hLHM1, BRCA1, VHL, PTEN, CDH1, CASP8, hTERTc and RASSF1c *in genome of control PC3 cells were found to be hypomethylated, and as expected, remained that way even after DNMT3b expression was curtailed (Figure-3). Also notable is the fact that gene promoters for *adenomatous polyposis coli (APC), retinoic acid receptor-beta (RAR-β), retinoblastoma-1 (Rb1) and tissue inhibitor of metalloprotein-3 (TIMP-3) *are differentially methylated on the two alleles. Strikingly, this experiment unequivocally demonstrated that from the 16 tumor suppressor genes that were scrutinized DNMT3b down-regulation caused specific loss of methylation at the promoters of *APC, RAR-β, and Rb1 *genes (Figure-3).

**Figure 4:**
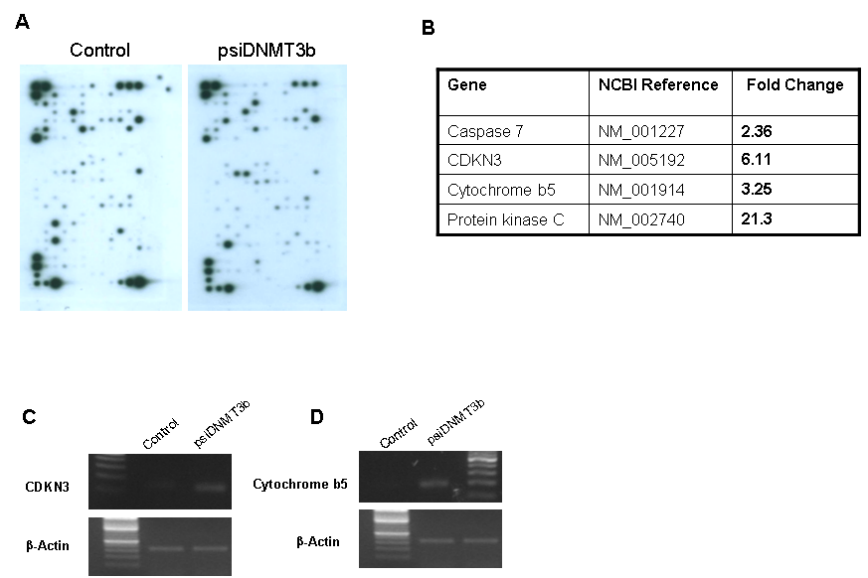
**Gene expression is impacted by DNMT3b silencing.** A. Expression profile obtained after mRNA was isolated from control and DNMT3b silenced PC3 cells, labeled and then hybridized to prostate cancer specific GE-array. B Results of gene expression profile. Only the genes whose expression increased in DNMT3b silenced cells 2-fold, relative to control cells, are listed. C and D. Expression of CDKN3 and cytochrome b5 was analyzed in DNMT3b silenced cells and controls by semi-quantitative RT-PCR.

To gain further insight into the consequences of DNMT3b silencing on the expression levels of various genes, a genechip array spotted with oligonucleotides representing a total of 288 (including 25 controls) genes found to be differentially expressed in prostate cancers was screened with biotin-labeled cRNA probes prepared from RNA that was purified from control and DNMT3b silenced PC3 cells (Figure-4A). The results of this experiment clearly showed that as compared to control cells, DNMT3b deficiency increased expression of *caspase 7, cytochrome b5, CDKN3 and protein kinase-C *by 2, 3, 6 and 21-fold, respectively (Figure-4B). The data from this experiment was further validated by performing semi-quantitative RT-PCR for *CDKN3 and cytochrome b5 *genes which showed significant up-regulation of these genes in DNMT3b silenced cell lines compared to the control cells (Figure-4C &4D).

## Discussion

In this report, we have shown that down-regulation of DNMT3b expression in PC3 cells is associated with loss of methylation at three (i.e., *APC, RAR-β and Rb1*) of the 16 gene promoters that were examined. Alleles for all three affected genes were found to be differentially methylated in mock-transfected PC3 cells, but DNMT3b silencing left both copies of each gene in a hypomethylated state. Additionally, from the 263 genes implicated in prostate cancer that were screened, four (i.e., *caspase-7, cdkn3, cytochrome b5 and protein kinase-C*) were found to be highly expressed (i.e., >2-fold) in DNMT3b silenced cells as compared to the controls; among the genes which were up-regulated, expression of *CDKN3 *increased 6-fold while that of *protein kinase-C *was found to be 21-fold higher. Although previous studies have shown that DNMT3b preferentially methylates satellite DNAs, this is the first report which demonstrates that DNMT3b also contributes to the specific silencing of various protein encoding genes.

Cell proliferation was dramatically affected by reduced DNMT3b levels and this was attributed to enhanced apoptosis suggesting that DNMT3b targets a set of genes whose products inhibit apoptosis. Interestingly, DNMT3b deficiency in PC3 cells did not affect their invasive potential but significantly compromised their ability to migrate indicating that the expression of genes which promote cell migration is influenced by DNMT3b mediated DNA methylation. Given that DNMT3b silencing promotes expression of proapoptotic genes such as *caspase-7 and cytochrome b5*, and negatively acting cell cycle regulators like *Rb1 and CDKN3 *explains why the host cells proliferate so poorly. This phenomenon is not restricted to PC3 cells only but has also been observed in cells obtained from human colorectal, breast and lung cancers [16,18].

The most striking aspect of our findings is that DNMT3b loss selectively led to the demethylation of *APC, Rb1 and RARβ *gene promoters. Moreover, even though the methylation status of the *caspase-7, CDKN3, cytochrome b5 and protein kinase-C *gene promoters was not determined, the fact that their expression levels increased in response to DNMT3b loss implies that these gene promoters are also targeted by this methylase. It is important to note that even though DNMT3b levels were reduced by 75%, the remaining 25% of methylase activity was insufficient to maintain the methylation status at several of the gene promoters.

Our findings are different from those reported by Rhee et al. who observed that DNMT3b knockout did not affect methylation of any of the examined genes but only caused demethylation of juxtacentromeric satellite-2 in colon cancer cells [17]. The impact of DNMT3b loss in PC3 cells appears to be more dramatic since the methylation and expression of many genes was affected. It should be noted however that PC3 cells contain moderate levels of DNMT1 and DNMT3a which are not affected when DNMT3b is silenced (data not shown); like PC3, colon cancer cells also express DNMT1 and DNMT3a. Since both DNMT1 and DNMT3a are unable to compensate for DNMT3b loss, it is plausible that colon carcinoma cells contain an unidentified component of the DNA methylation pathway which is absent in PC3 cells.

Only a small number of genes were included in this study and the one reported by Rhee et al. To clearly discern the differences that exist between DNMT3b silenced PC3 and colon cancer cells, will require detailed gene expression as well as whole genome analysis in order to identify the sets of gene loci that are most impacted by DNMT3b silencing.

DNA methylation was once assumed to be a simple process dependent entirely on DNMT1, DNMT3a and DNMT3b. However a number of biochemical, genetic and cell-based studies have found that the interplay between these enzymes is complex and most likely dependent on additional proteins. By demonstrating that silencing of DNMT3b in prostate derived cells has a more dramatic effect than its absence in colon carcinoma cells, this work also supports the notion that other yet unidentified factors are likely to influence DNMT3b activity.

## Conclusion

Taken together our data demonstrates that silencing DNMT3b expression causes hypomethylation of specific sets of gene promoters and increases expression of distinct set of genes. Phenotypic characterization of DNMT3b silenced PC3 cells showed that they grew poorly and had reduced migratory potential as compared to the control cells.

## Abbreviations

DNMT3b: DNA methyltransferase 3b; CDKN3: Cyclin dependent kinase inhibitor 3; RB1: Retinoblastoma protein; APC: Adenomatous polyposis coli; RAR-β: Retinoic acid receptor beta.

## Competing interests

The authors declare that they have no competing interests.

## Authors' contributions

AY carried out all experimental work. SAQ conceived ideas, analyzed data and wrote manuscript. RQ contributed in setting up invasion assays. FA analyzed results and evaluated manuscript. All authors read and approved the final manuscript.
